# Contrasting evolutionary patterns between two haplogroups of *Haematobia exigua* (Diptera: Muscidae) from the mainland and islands of Southeast Asia

**DOI:** 10.1038/s41598-017-05921-w

**Published:** 2017-07-19

**Authors:** Van Lun Low, Tiong Kai Tan, Batah Kunalan Prakash, Wei Yin Vinnie-Siow, Sun Tee Tay, Roungthip Masmeatathip, Upik Kesumawati Hadi, Yvonne Ai Lian Lim, Chee Dhang Chen, Yusoff Norma-Rashid, Mohd Sofian-Azirun

**Affiliations:** 1 Tropical Infectious Diseases Research and Education Centre (TIDREC), University of Malaya, Kuala Lumpur, Malaysia; 2 Department of Parasitology, Faculty of Medicine, University of Malaya, Kuala Lumpur, Malaysia; 3 Biodiversity Research Center, Academia Sinica, Taipei, Taiwan; 4 Institute of Biological Sciences, Faculty of Science, University of Malaya, Kuala Lumpur, Malaysia; 5 Department of Medical Microbiology, Faculty of Medicine, University of Malaya, Kuala Lumpur, Malaysia; 6 Department of Entomology, Faculty of Agriculture, Kasetsart University, Kamphaeng Saen, Thailand; 7 Department of Animal Infectious Diseases and Veterinary Public Health, Faculty of Veterinary Medicine, Bogor Agricultural University, Bogor, Indonesia

## Abstract

Uncovering the hidden diversity and evolutionary history of arthropods of medico-veterinary importance could have significant implications for vector-borne disease control and epidemiological intervention. The buffalo fly *Haematobia exigua* is an obligate bloodsucking ectoparasite of livestock. As an initial step towards understanding its population structures and biogeographic patterns, we characterized partial cytochrome c oxidase subunit I (COI) and cytochrome b (Cytb) sequences of *H. exigua* from three distinct geographic regions in Southeast Asia. We detected two distinct mitochondrial haplogroups of *H. exigua* in our surveyed geographic regions. Haplogroup I is widespread in the Southeast Asian mainland whereas haplogroup II is generally restricted to the type population Java Island. Both haplogroups were detected co-occurring on Borneo Island. Additionally, both haplogroups have undergone contrasting evolutionary histories, with haplogroup I exhibited a high level of mitochondrial diversity indicating a population expansion during the Pleistocene era dating back to 98,000 years ago. However, haplogroup II presented a low level of mitochondrial diversity which argues against the hypothesis of recent demographic expansion.

## Introduction

Empirical knowledge of population genetics and the evolutionary background of arthropod vectors can be used to understand vector and pathogen interactions, anticipate risks, and develop control strategies^1^. Particularly, the discovery of distinct genetic structures has immense epidemiological implications because different populations of vectors may present variable susceptibility levels to pathogen infections^2, 3^.

The buffalo fly *Haematobia exigua* is an obligate bloodsucking ectoparasite of livestock, specifically cattle and buffalo in the Australasian and Oriental regions^4, 5^. To better understand this notorious pest at a molecular level, several genetic approaches have been conducted to resolve the taxonomic boundaries between *H. exigua* and its congener *H. irritans* ^5–7^. While *H. exigua* has been recognized as a distinct species, its population genetics and evolutionary history have not been characterized so far.

The Southeast Asia is home to the type locality of *H. exigua* where this species was first described on the island of Java, Indonesia^4^. In the past few decades, the demographic history of certain organisms in Southeast Asia has been a topic of intense interest for many researchers^8–12^. During the last glacial period, several regions such as northern and eastern Borneo, northern and western Sumatra, and the Mentawai islands in South East Asia were the refugium sites for some organisms while the mainland areas—Thailand and peninsular Malaysia were severely affected by the Pleistocene drought^9^. Nevertheless, there is convincing evidence for the existence of glacial refugia on the mainland^10^. However, the responses of *H. exigua* in Southeast Asia to these major historical climate changes, are yet to be investigated.

**Figure 1:**
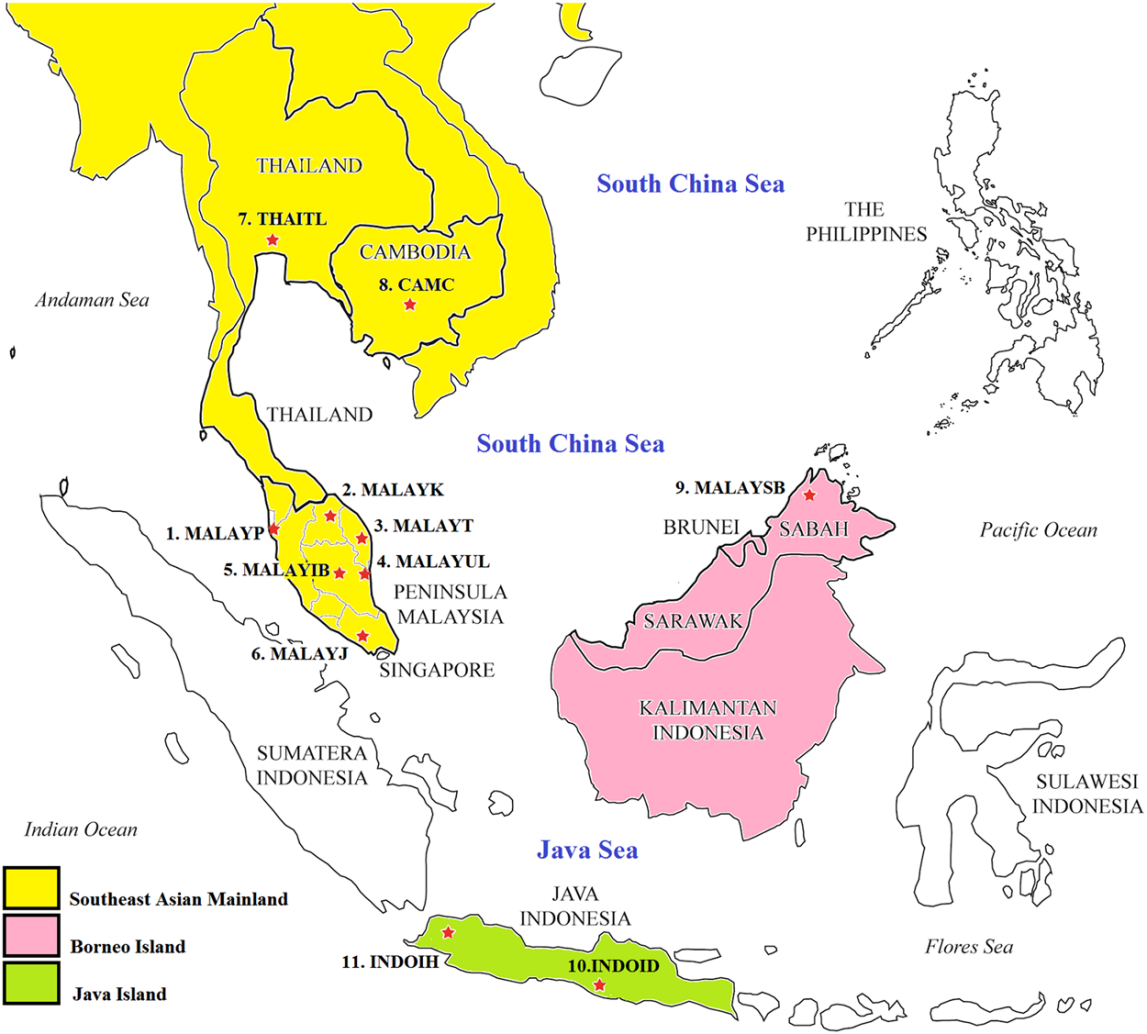
An outline map of Southeast Asia depicting sampling sites of *Haematobia exigua* used in this study. The map was created with QGIS software 2.18.3 (http://www.qgis.org/pl/site/) and modified by VLL with help from Z. Mustafa using Adobe Photoshop CS4.

Accordingly, we sampled *H. exigua* from four countries across a geographic range of over 2,600 km in Southeast Asia (Table 1 and Fig. 1) to determine if this species consists of more than one genetically distinct taxon, and further characterize its population genetic structure and demographic history.

**Table 1:** Sampling sites and distribution of *Haematobia exigua* haplogroups in Southeast Asia.

Sampling site (Geographic region)	Country	Sample code (n)	Haplogroup
*Southeast Asian Mainland*			
1. Bukit Tengah, Penang (North peninsula)	Malaysia	MALAYP (22)	I
2. Tanah Merah, Kelantan (East peninsula)	Malaysia	MALAYK (15)	I
3. Kuala Berang, Terengganu (East peninsula)	Malaysia	MALAYT (17)	I
4. Kuantan, Pahang (East peninsula)	Malaysia	MALAYUL (3)	I
5. Jerantut, Pahang (West peninsula)	Malaysia	MALAYIB (3)	I
6. Air Hitam, Johore (South peninsula)	Malaysia	MALAYJ (17)	I
7. Chatuchak, Bangkok (Central Thailand)	Thailand	THAITL (20)	I
8. Por Sen Chey, Phnom Penh (South-Central Cambodia)	Cambodia	CAMC (5)	I
*Borneo Island*			
9. Ranau, Sabah (North Borneo)	Malaysia	MALAYSB (20)	I, II
*Java Island*			
10. Bleberan, Yogyakarta (Central Java)	Indonesia	INDOID (15)	II
11. Cibungbulang, Bogor (West Java)	Indonesia	INDOIH (25)	II

## Results

### Molecular markers

Of several mitochondrial and nuclear genes tested in our previous study^6^, the mitochondrial cytochrome c oxidase subunit I (COI), cytochrome b (Cytb) and NADH dehydrogenase subunit 5 (ND5) genes were found to be more variable and informative in resolving the intra- and inter-specific relationships between *H. exigua* and *H. irritans*. These three genes were therefore adopted in the present study for a preliminary assessment of the genetic divergence of *H. exigua* collected from various geographic regions. However, the ND5 gene appeared to be insensitive at a species level because it provided a less-resolved topology that was incongruent with the COI and Cytb genes. Hence, further analyses were performed by using the latter two genes as molecular markers in this study.

### Phylogenetic reconstruction

**Figure 2:**
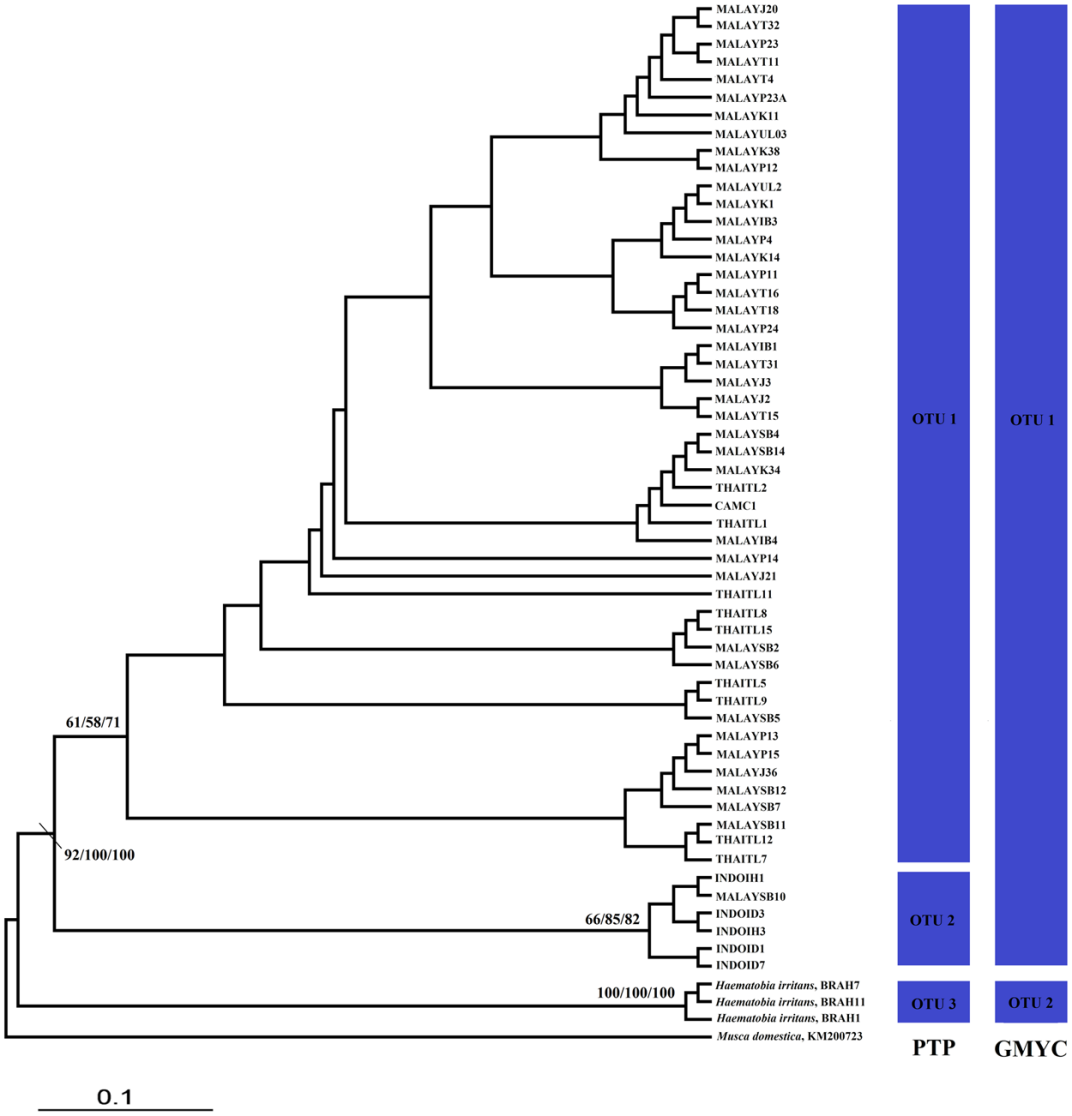
Bootstrap [maximum likelihood (ML)/maximum parsimony (MP)/ neighbour-joining (NJ)] values are shown on the branches. Values less than 50 are not shown. The scale bar represents 0.1 substitutions per nucleotide position. The blue columns on the right show numbers of operational taxonomic units (OUTs) identified by the species delimitation analyses.

Maximum likelihood (ML), maximum parsimony (MP), and neighbor-joining (NJ) phylogenetic analyses led to similar hypotheses for the evolutionary assemblages of *Haematobia* taxa. The ML tree for the concatenated sequences of COI and Cytb is shown in Fig. 2. The phylogenetic tree revealed two intra-specific lineages (hereafter named haplogroups) of *H. exigua*: haplogroup I is composed of populations from the Southeast Asian mainland (Cambodia, peninsular Malaysia and Thailand), and Borneo; whereas haplogroup II includes populations from the islands of Java and Borneo. Interestingly, both haplogroups were detected in our samples from Borneo, albeit with only one specimen assigned to haplogroup II. The congener *H. irritans* formed a well-supported basal assemblage (Table 1 and Fig. 2).

### Sequence-based species delimitation

DNA-based species delimitation based on poisson tree processes (PTP) and generalized mixed yule-coalescent (GMYC) analyses revealed discordant results with respect to the numbers of operational taxonomic units (OTUs) within *Haematobia* spp. PTP analysis differentiated *H. exigua* into two distinct OTUs, corresponding to the identified haplogroups. However, GMYC analysis recognized all *H. exigua* populations as single OTU. Both analyses identified *H. irritans* as a valid species (Fig. 2).

### Isolation by distance, genetic differentiation and gene flow

**Figure 3:**
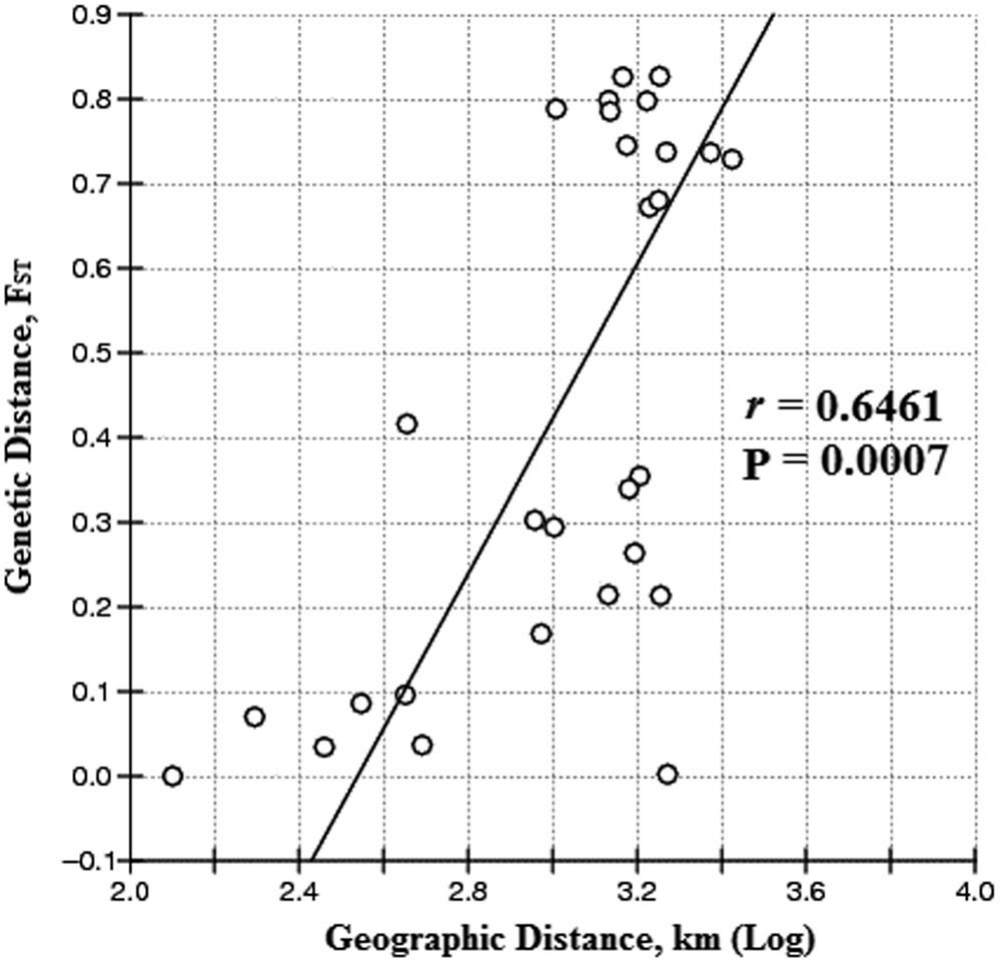
A Mantel test for correlation between genetic distance and geographic distance.

The Mantel test for isolation by distance suggested a significant correlation between genetic distance and geographic distance (*r* = 0.6461; P = 0.0007) (Fig. 3). Comparable results were also observed when two populations from Java were excluded from the analysis (*r* = 0.6637; P = 0.0072) (data not shown).

The study revealed a relatively moderate level of genetic differentiation (F_ST_ = 0.198) but there was a high gene flow (Nm = 2.03) between populations from the mainland and Borneo. In contrast, high levels of genetic differentiation and low gene flow were identified between populations from the mainland and Java (F_ST_ = 0.736, Nm = 0.18), as well as between Borneo and Java (F_ST_ = 0.655, Nm = 0.26) (Table 2).

**Table 2:** Genetic differentiation, F_ST_ and gene flow, Nm (in brackets) among *Haematobia exigua* in the Southeast Asian mainland, Borneo Island and Java Island.

	1	2	3
1. Southeast Asian mainland	–		
2. Borneo Island	0.198* (2.03)	–	
3. Java Island	0.736* (0.18)	0.655* (0.26)	–

^*^P < 0.001.

### Genetic divergence

Given the enormous difference in sample size between haplogroups I and II, we constructed a rarefaction curve of observed haplotypes to determine the effect of sampling on their genetic diversity. As far as the sample size of haplogroup II is concerned, a saturation point was observed from the curve (data not shown), suggesting that sampling was complete and additional sampling was not required. Accordingly, the genetic divergences and demographic histories of the two haplogroups were compared in the subsequent analyses.

The inter-specific distances based on the concatenated sequences ranged from 0.06 to 2.84%. *Haematobia exigua* haplogroup I differed from haplogroup II by 0.12 to 0.95% and from *H. irritans* by 2.37 to 2.84%. Haplogroup II differed from *H. irritans* by 2.31 to 2.67%. The intra-specific distances ranged from 0.06 to 0.71% (Table 3).

**Table 3:** Intra-and inter-specific uncorrected *p* genetic distances (%) among three taxa of *Haematobia* flies.

	1	2	3
1. *H. exigua* haplogroup I	0.06–0.71		
2. *H. exigua* haplogroup II	0.12–0.95	0.06–0.36	
3. *H. irritans*	2.37–2.84	2.31–2.67	0.12

**Figure 4:**
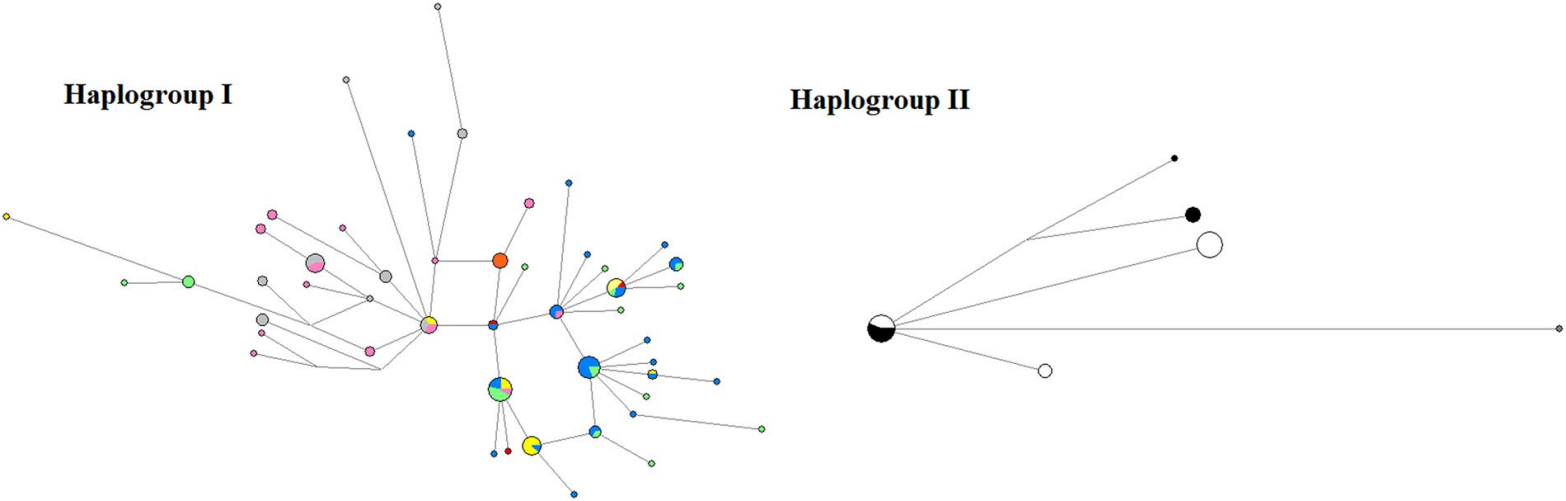
Median joining haplotype network of *Haematobia exigua* haplogroups in Southeast Asia. Each haplotype is represented by a circle. Relative sizes of the circles indicate haplotype frequency. Circles of the same colour represent haplotypes from the same population (green = North peninsula, blue = East peninsula, red = West peninsula, yellow = South peninsula, pink = Central Thailand, orange = South-Central Cambodia, grey = North Borneo, black = Central Java, and white = West Java).

Of the 121 individuals of *H. exigua* haplogroup I which were analyzed in this study, 49 distinct haplotypes were discovered, 11 of which were shared among populations. The haplotypes were well dispersed across all study sites. For haplogroup II, a total of six distinct haplotypes were identified from 41 individuals, and only one was shared between two populations. We found a high haplotype diversity (0.964) but low nucleotide diversity (0.002) in haplogroup I, whereas low haplotype (0.002) and nucleotide (0.001) diversities were found in haplogroup II (Table 4 and Fig. 4).

**Table 4:** Number of haplotype (h), haplotype diversity (Hd), nucleotide diversity (Pi), Tajima’s D (D), Fu’s Fs (Fs) and Fu & Li’s D* (D*) tests based on haplogroups of *Haematobia exigua*.

Haplogroup	n	h	Hd	Pi	D	Fs	D*
I	121	49	0.964	0.002	−1.540*	−34.533**	−3.285*
II	41	6	0.002	0.001	−0.654	−0.556	−1.524

^*^P < 0.05, **P < 0.001.

### Demographic history

**Figure 5:**
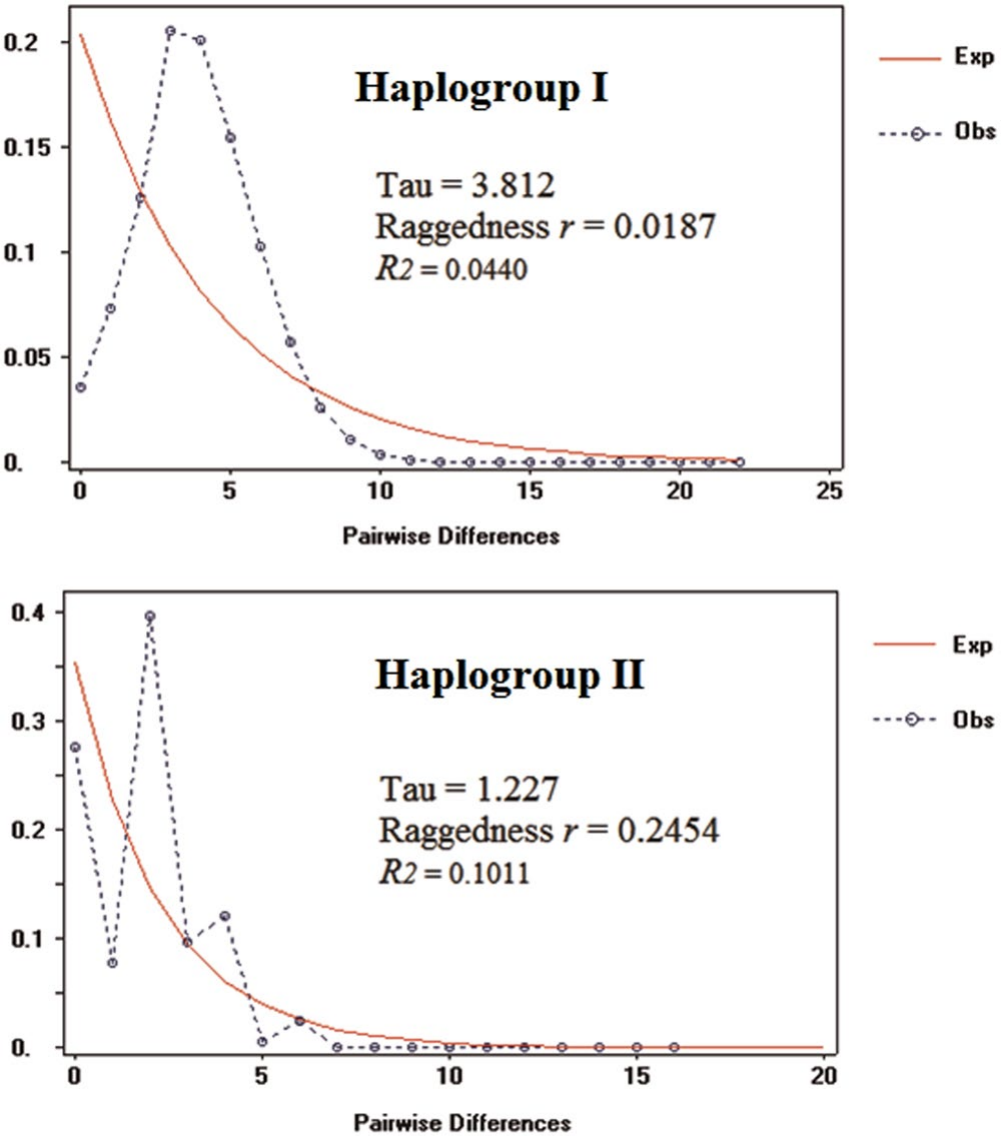
Observed and expected mismatch distributions for *Haematobia exigua* haplogroups in Southeast Asia.

Our data suggests that haplogroup I has undergone a recent population expansion, as evidenced by the “star-like” network (Fig. 4). The unimodal mismatch distribution, low values of the Raggedness index (*r* = 0.0187, P > 0.05) and R_2_ statistic (R_2_ = 0.0440, P < 0.05) from mismatch distribution tests (Fig. 5), along with the significant negative values of Tajima’s D, Fu’s Fs and Fu & Li’s D* tests (Table 4), further supported our hypothesis of population expansion in *H. exigua* haplogroup I from the mainland of Southeast Asia and Borneo. The expansion time was estimated to be 98,000 years ago.

By contrast, haplogroup II revealed a non-significant R_2_ statistic value (R_2_ = 0.1011), a high Raggedness index value (*r* = 0.2454, P > 0.05) (Fig. 5), and non-significant values of all three neutrality tests (Table 4), rejecting the hypothesis of population expansion. The multimodal mismatch distribution and low haplotype and nucleotide diversities are consistent with a recent bottleneck effect.

## Discussion

### Taxonomic status of *Haematobia* taxa

The taxonomic status of *Haematobia* taxa has long been questioned because of their similar morphological characteristics that cannot easily be distinguished^6, 13^. Although the morphological differences between both taxa are minor, *H. exigua* differs from *H. irritans* by the presence of 4 to 6 distinctive long hairs with curled tips on the segments of the male’s hind tarsus^13^. In addition, previous studies elsewhere have provided genetic^5–7^ and chemotaxonomic^14^ evidence to support their recognition as separate species, rather than as subspecies. Our present DNA-based species delimitation based on PTP and GMYC analyses unambiguously recognized *H. irritans* as a distinct OTU, further supporting its full species status.

Assessment of the species status of *H. exigua* is complicated by the presence of two haplogroups in Southeast Asia. As shown in the phylogenetic tree, both haplogroups were not well supported (<90%), but lower bootstrap values are expected within a species or closely related taxa^15, 16^. Nevertheless, two species delimitation analyses revealed different taxonomic entities within *H. exigua*. PTP analysis recognized haplogroups I and II as two distinct OTUs, however, GMYC treated both haplogroups as a single OTU. If both haplogroups deserve species status, their genetic divergence should be taken into consideration such as is comparable to the divergence observed between closely related and uncontroversial species pairs^17^. The genetic distances between two haplogroups, however, were relatively low (0.12–0.95%), and were actually much lower than the differences between *H. exigua* and *H. irritans* (2.31–2.84%). Given these relatively slight differences, we tentatively conclude that *H. exigua* represents a single species, rather than two distinct species.

### Genetic diversity

Haplogroup I exhibited higher genetic diversity than did haplogroup II. Haplogroup I revealed 49 unique haplotypes (out of 121 individuals), high haplotype diversity (0.964), and genetic distances up to 0.71%. In contrast, haplogroup II revealed only six unique haplotypes (out of 41 individuals), with extremely low haplotype diversity (0.002) and 0.36% genetic distances. The diversity of haplogroup I on the Southeast Asian mainland was greater than that of haplogroup II on Java Island. Our results are consistent with previous findings, in which a common simulid black fly *Simulium nobile* De Mejiere also demonstrated similar genetic pattern on the mainland and islands in Southeast Asia^18^. Perhaps, a lack of genetic diversity is an ordinary characteristic of island populations, possibly because of the bottleneck effect, genetic drift, and isolation^19, 20^.

### Isolation by distance, genetic differentiation and gene flow

The present study showed that geographic distance could have significant effects on the genetic structure of *H. exigua* in Southeast Asia. A Mantel test detected a pattern of isolation by distance, revealing a significant relationship between pairwise genetic and geographic distances for the studied regions.

Java and Borneo islands are substantially segregated from the Southeast Asian mainland by the South China Sea and Java Sea, and this could be a factor associated with intra-specific genetic discontinuities^20, 21^. The Java Sea is likely to be a barrier to gene flow between *H. exigua* on the mainland and Java, and between Java and Borneo. This hypothesis is supported by the high genetic differentiation and low gene flow in our datasets. Indeed, the oceanographic barrier to dispersal and gene flow has been suggested as a key factor driving diversification in several insects^10, 22^. Nevertheless, the South China Sea did not have a limiting effect on gene flow for *H. exigua* on the mainland and Borneo. The observed moderate genetic differentiation could be due to the recurrent gene flow across both geographic regions or the sharing of a recent common history^23^ (see below). On the contrary, we certainly cannot exclude the possibility of gene flow via human-mediated transportation of fly-infested animals. Previous studies elsewhere have suggested that the natural dispersal ability of livestock ectoparasites could occur in parallel with human-mediated dispersal, leading to genetic admixture and high gene flow^24, 25^.

### Biogeography and evolutionary histories of the haplogroups of *H. exigua*

To examine the response of *H. exigua* to historical climate change events, we conducted a series of demographic analyses based on mitochondrial DNA, and we detected remarkable differences between the two haplogroups of *H. exigua* based on multiple lines of evidence. A “star-like” network, a unimodal mismatch distribution, low values of the Raggedness index and the R_2_ statistic from mismatch distribution tests, along with the significant negative values of neutrality tests suggested that haplogroup I has a historical expansion pattern through the late Pleistocene dating back to 98,000 years ago. In the past few decades, several insects in Southeast Asia have also demonstrated demographic expansion during the Quaternary glaciation period^8, 10–12, 26, 27^. The black fly *S. angulistylum* Takaoka & Davies in Thailand has the oldest demographic expansion for insects dating back up to 930,000 years ago^28^, whereas the Thai *S. nododum* Puri has the youngest history dating back to only 2,600–5,200 years ago^29^.

By contrast, haplogroup II has rejected the hypothesis of recent demographic expansion, as evidenced by all demographic analyses. Additionally, we also observed a multimodal mismatch distribution and this pattern could be an indicator of a recent bottleneck^30^. An extremely low level of genetic diversity, with low haplotype diversity and nucleotide diversities are also associated with bottleneck effects^31, 32^, thus lending support to our hypothesis of a recent bottleneck in these populations.

## Methods

### Ethical approval

All experiments were performed in accordance with relevant guidelines and regulations of the University of Malaya. The research protocols were regulated and approved by the University of Malaya. *Haematobia* taxa are neither protected nor endangered species, hence, no specific permits were required for this study.

### Taxon sampling and species identification

A total of 162 individuals of *H. exigua* were collected from cattle farms in four countries across a geographic range of over 2,600 km in Southeast Asia including the mainland (Cambodia, Thailand and West Malaysia), Borneo (East Malaysia), and Java (Indonesia) (Table 1 and Fig. 1). Given the relatively small sample size of haplogroup II, we performed a rarefaction analysis using R 3.2.1 to determine the effect of sampling on genetic diversity.

The congener *H. irritans* analyzed from our previous study^6^ was also included for phylogenetic inference. Species identification was performed using morphological keys^13^. The representative specimens were deposited at the Tropical Infectious Diseases Research and Education Centre (TIDREC), University of Malaya, Malaysia.

### DNA isolation, amplification, and sequencing

Total DNA was isolated from each adult specimen, using the i-genomic CTB DNA Extraction Mini Kit (iNtRON Biotechnology Inc., Seongnam, South Korea). DNA was eluted in 50 μL of elution buffer and stored at −20 °C until further analyses. DNA amplifications were performed by polymerase chain reaction (PCR) using an Applied Biosystems Veriti 96-Well Thermal Cycler (Applied Biosystems, Inc., Foster City, CA, USA).

Two sets of primers were used to amplify the COI and Cytb gene regions as described in Low *et al*.^6^. The PCR products were then sequenced in forward and reverse directions using BIG DYE Terminator v3.1 by an ABI 3730XL Genetic Analyzer (Applied Biosystems Inc., Foster City, CA, USA). DNA sequences generated in this study are accessible from the National Center for Biotechnology Information (NCBI) GenBank under accession numbers KU599938-KU599978 for COI and KU599979-KU600012 for Cytb.

### Sequence alignment and partition homogeneity test

COI and Cytb sequences were assembled using ChromasPro 1.7.6 (Technelysium Pty Ltd., Australia) and edited using BioEdit 7.0.9.0^33^. To examine whether each COI and Cytb dataset could be concatenated into a single dataset, statistical congruence was calculated using a partition homogeneity test implemented in PAUP 4.0b10^34^. No significant differences were found among separate gene regions (P = 1.00). Hence, COI and Cytb sequences were concatenated for all subsequent data analyses.

### Phylogenetic reconstruction

Representative haplotype sequences were subjected to phylogenetic analyses. The ML analysis was performed on an on-line web-based server PhyML 3.0^35^. An automatic model selection was implemented based on the Akaike information criterion (AIC). The best-fit model was the general time-reversible (GTR) model with a proportion of invariable sites of 0.676 and with a gamma shape parameter of 0.887. The MP tree was constructed using MEGA 6.0^36^ with 10 random sequence additions and tree bisection reconnection (TBR) branch swapping. The MP bootstrap values were computed with 1000 resamplings. The NJ tree was constructed using PAUP 4.0b10. NJ bootstrap values were estimated using 1000 replicates with Kimura’s two-parameter model of substitution (K2P distance). The house fly, *Musca domestica* Linnaeus (KM200723) was used as an outgroup.

### Sequence-based species delimitation

To compare the discriminatory power with the species boundaries defined by the conventional phylogenetic analyses, we performed two species delimitation methods: poisson tree processes (PTP) and generalized mixed yule coalescent (GMYC). PTP analysis was performed with the bPTP web-server^37^. A maximum likelihood solution (PTP_ML) model was applied, and analysis was run with 100,000 Markov chain Monte Carlo (MCMC) generations, thinning set to 100 and burnin at 0.1.

For GMYC analysis, the ultrametric tree was generated from the representative haplotypes in BEAST 1.8.236 using a relaxed lognormal clock, coalescent (constant size) prior and GTR + I + G model of DNA substitution. The analysis was run for 20 million generations, with a sampling frequency of every 100 generations. The output tree was analyzed in TreeAnnotator 1.8.2 with a 10% burn-in. The data were analyzed using a single threshold model in the software package SPLITS^38^ available in R 3.2.1.

### Isolation by distance, genetic differentiation, and gene flow

Levels of genetic differentiation and gene flow were assessed using DnaSP 5.0^39^. The correlation between genetic distance (F_ST_) and geographic distance (km) was examined using a Mantel test^40^ implemented with the program Isolation by Distance Web Service 3.23^41^.

### Genetic divergence and demographic history

To assess levels of variation among the delimited taxa based on PTP and GMYC analyses, uncorrected *p* pairwise genetic distances were estimated using PAUP 4.0b10.

Haplotype and nucleotide diversities were assessed using DnaSP 5.0. Haplotype network reconstructions based on haplogroups were performed using a median-joining algorithm^42^ in the program Network 4.6.

To test for population equilibrium and signature of population expansion, Tajima’s D^43^, Fu’s Fs^44^, Fu & Li’s D*^45^, Harpending’s raggedness index^46^, R_2_ statistic of Ramos-Onsins & Rozas^47^, and mismatch distribution tests were performed using DnaSP 5.0. If an expansion event has occurred, the time since expansion can be calculated using a mismatch calculator^48^. The expansion time was estimated according to a divergence rate of 2.3% per 1,000,000 years for insect mitochondrial DNA^49^.

## References

[1] Goubert C, Minard G, Vieira C, Boulesteix M (2016). Population genetics of the Asian tiger mosquito *Aedes albopictus*, an invasive vector of human diseases. Heredity.

[2] Burlini L, Teixeira KRS, Szabó PJM, Famadas KM (2010). Molecular dissimilarities of *Rhipicephalus sanguineus* (Acari: Ixodidae) in Brazil and its relation with samples throughout the world: is there a geographical pattern?. Exp. Appl. Acarol..

[3] Demoner Lde C (2013). Investigation of tick vectors of Hepatozoon canis in Brazil. Ticks Tick Borne Dis..

[4] Mackerras IM (1933). The taxonomy of *Lyperosia exigua* De Meijere (Diptera, Muscidæ). Ann. Mag. Nat. Hist..

[5] Iwasa M, Ishiguro N (2010). Genetic and morphological differences of *Haematobia irritans* and *H. exigua*, and molecular phylogeny of Japanese Stomoxyini flies (Diptera, Muscidae). Med. Entomol. Zool..

[6] Low VL (2014). Use of COI, CytB and ND5 genes for intra- and inter-specific differentiation of *Haematobia irritans* and *Haematobia exigua*. Vet. Parasitol..

[7] Changbunjong T, Weluwanarak T, Samung Y, Ruangsittichai J (2016). Molecular identification and genetic variation of hematophagous flies, (Diptera: Muscidae: Stomoxyinae) in Thailand based on cox1 barcodes. J. Asia Pac. Entomol..

[8] Walton C (2000). Population structure and population history of *Anopheles dirus* mosquitoes in Southeast Asia. Mol. Biol. Evol..

[9] Gathorne-Hardy FJ, Syaukani, Davies RG, Eggleton P, Jones DT (2002). Quaternary rainforest refugia in south-east Asia: using termites (Isoptera) as indicators. Biol. J. Linn. Soc..

[10] Pramual P, Kuvangkadilok C, Baimai V, Walton C (2005). Phylogeography of the black fly *Simulium tani* (Diptera: Simuliidae) from Thailand as inferred from mtDNA sequences. Mol. Ecol..

[11] Low VL (2014). Mitochondrial DNA markers reveal high genetic diversity but low genetic differentiation in the black fly *Simulium tani* Takaoka & Davies along an elevational gradient in Malaysia. PLoS ONE.

[12] Low VL (2017). Pleistocene demographic expansion and high gene flow in the Globe Skimmer dragonfly *Pantala flavescens* Fabricius (Odonata: Libellulidae) in Peninsular Malaysia. Zool. Anz..

[13] Kano R, Shinonaga S, Hasegawa T (1972). On the specific name of *Haematobia* (Diptera Muscidae) from Japan. Jpn. J. Sanit. Zool..

[14] Urech R, Brown GW, Moore CJ, Green PE (2005). Cuticular hydrocarbons of buffalo fly, *Haematobia exigua*, and chemotaxonomic differentiation from horn fly, *H. irritans*. J. Chem. Ecol..

[15] Kerr KCR (2007). Comprehensive DNA barcode coverage of North American birds. Mol. Ecol. Notes.

[16] Stoneking, M. An Introduction to Molecular Anthropology (Wiley-Blackwell, United States, 2016).

[17] Ron SR, Santos JC, Cannatella DC (2006). Phylogeny of the tungara frog genus *Engystomops* (=*Physalaemus pustulosus* species group; Anura: Leptodactylidae). Mol. Phylogenet. Evol..

[18] Low VL (2016). Three taxa in one: cryptic diversity in the black fly *Simulium nobile* (Diptera: Simuliidae) in Southeast Asia. J. Med. Entomol..

[19] Eldridge MDB, Kinnear JE, Zenger KR, McKenzie LM, Spencer PBS (2004). Genetic diversity in remnant mainland and “pristine” island populations of three endemic Australian macropodids (Marsupialia): *Macropus eugenii*, *Lagorchestes hirsutus* and *Petrogale lateralis*. Conserv. Genet..

[20] Boessenkool S, Taylor SS, Tepolt CK, Komdeur J, Jamieson IG (2007). Large mainland populations of South Island robins retain greater genetic diversity than offshore island refuges. Conserv. Genet..

[21] Castella V (2000). Is the Gibraltar Strait a barrier to gene flow for the bat *Myotis myotis* (Chiroptera: Vespertilionidae)?. Mol. Ecol..

[22] Andrews KR, Norton EL, Fernandez-Silva I, Portner E, Goetze E (2014). Multilocus evidence for globally distributed cryptic species and distinct populations across ocean gyres in a mesopelagic copepod. Mol. Ecol..

[23] Templeton AR (1998). Nested clade analyses of phylogeographic data: testing hypotheses about gene flow and population history. Mol. Ecol..

[24] Busch JD (2014). Widespread movement of invasive cattle fever ticks (*Rhipicephalus microplus*) in southern Texas leads to shared local infestations on cattle and deer. Parasit Vector.

[25] Low VL (2015). Molecular characterisation of the tick *Rhipicephalus microplus* in Malaysia: new insights into the cryptic diversity and distinct genetic assemblages throughout the world. Parasit Vector.

[26] Pramual P, Kongim B, Nanork P (2011). Phylogeography of *Simulium siamense* Takaoka and Suzuki complex (Diptera: Simuliidae) in Thailand. Entomol. Sci..

[27] Hughes, J., Schmidt, D., McLean, A. & Wheatley, A. *Population genetic structure in stream insects: what have we learned*? *Aquatic Insects: Challenges to Populations*. Lancaster, J. & Briers R. A. (eds) 268–288 (CABI Publishing, UK, 2008).

[28] Pramual P, Kuvangkadilok C (2012). Integrated cytogenetic, ecological, and DNA barcode study reveals cryptic diversity in *Simulium* (*Gomphostilbia*) *angulistylum* (Diptera: Simuliidae). Genome.

[29] Chaiyasan P, Pramual P (2016). Population genetic structure and demographic history of the black fly vector, *Simulium nodosum* in Thailand. Med. Vet. Entomol..

[30] Liao PC (2010). Historical spatial range expansion and a very recent bottleneck of *Cinnamomum kanehirae* Hay. (Lauraceae) in Taiwan inferred from nuclear genes. BMC Evol. Biol..

[31] Nei M, Maruyama T, Chakraborty R (1975). The bottleneck effect and genetic variability in populations. Evol..

[32] Low VL (2014). Mitochondrial DNA analyses reveal low genetic diversity in *Culex quinquefasciatus* from residential areas in Malaysia. Med. Vet. Entomol..

[33] Hall TA (1999). BioEdit: a user-friendly biological sequence alignment editor and analysis program for Windows 95/98/NT. Nucleic Acids Symp. Ser..

[34] Swofford, D. L. PAUP: Phylogenetic Analysis Using Parsimony (and Other Methods), version 4.0 b10 (2002).

[35] Guindon S (2010). New algorithms and methods to estimate maximum-likelihood phylogenies: assessing the performance of PhyML 3.0. Syst. Biol..

[36] Tamura K, Stecher G, Peterson D, Filipski A, Kumar S (2013). MEGA6: Molecular Evolutionary Genetics Analysis version 6.0. Mol. Biol. Evol.

[37] Zhang J, Kapli P, Pavlidis P, Stamatakis A (2013). A general species delimitation method with applications to phylogenetic placements. Bioinformatics.

[38] Ezard, T., Fujisawa, T. & Barraclough, T. Splits: SPecies’ LImits by Threshold Statistics R package http://r-forge.rproject.org/projects/splits/ (2009).

[39] Librado P, Rozas J (2009). DnaSP v5: a software for comprehensive analysis of DNA polymorphism data. Bioinformatics.

[40] Mantel N (1976). The detection of disease clustering and a generalized regression approach. Cancer Res..

[41] Jensen JL, Bohonak AJ, Kelley ST (2005). Isolation by distance, web service. BMC Genet..

[42] Bandelt HJ, Forster P, Röhl A (1999). Median-joining networks for inferring intraspecific phylogenies. Mol. Biol. Evol..

[43] Tajima F (1989). Statistical method for testing the neutral mutation hypothesis by DNA polymorphism. Genet.

[44] Fu YX (1997). Statistical tests of neutrality of mutations against population growth, hitchhiking and background selection. Genet..

[45] Fu YX, Li WH (1993). Statistical tests of neutrality of mutations. Genet..

[46] Harpending HC (1994). Signature of ancient population growth in a low-resolution mitochondrial DNA mismatch distribution. Hum. Biol..

[47] Ramos-Onsins SE, Rozas J (2002). Statistical properties of new neutrality tests against population growth. Mol. Biol. Evol..

[48] Schenekar T, Weiss S (2011). High rate of calculation errors in mismatch distribution analysis results in numerous false inferences of biological importance. Heredity.

[49] Brower AVZ (1994). Rapid morphological radiation and convergence among races of the butterfly *Heliconius erato*, inferred from patterns of mitochondrial DNA evolution. Proc. Natl. Acad. Sci. USA.

